# Exploring the liver–kidney axis in the Ghanaian population: the RODAM prospective study

**DOI:** 10.7189/jogh.16.04252

**Published:** 2026-07-24

**Authors:** Muhulo Muhau Mungamba, Felix Patience Chilunga, Eva L van der Linden, Erik Beune, Peter Henneman, Charles Frederick Hayfron-Benjamin, Karlijn Anna Catharina Meeks, Samuel Nkansah Darko, Sampson Twumasi-Ankrah, Ellis Owusu-Dabo, Adriaan Georgius Holleboom, Liffert Vogt, Bert-Jan van den Born, Benedicta Ngwenchi Nkeh-Chungag, Charles Agyemang

**Affiliations:** 1 Department of Public and Occupational Health, Amsterdam University Medical Center, Amsterdam, The Netherlands; 2 Department of Human Biology, Nelson Mandela Drive, Faculty of Health Sciences, Walter Sisulu University, Mthatha, South Africa; 3 Department of Human Genetics and Epigenetics, Amsterdam University Medical Centers, Amsterdam, The Netherlands; 4 Department of Physiology and Anaesthesia/Critical Care, University of Ghana Medical School / Korle Bu Teaching Hospital, Accra, Ghana; 5 Division of Endocrinology, Diabetes and Nutrition, University of Maryland School of Medicine, Baltimore, USA; 6 Department of Molecular Medicine, Kwame Nkrumah University of Science and Technology, Kumasi, Ghana; 7 Department of Statistics and Actuarial Science, Kwame Nkrumah University of Science and Technology, Kumasi, Ghana; 8 Department of Global and International Health, Kwame Nkrumah University of Science and Technology, Kumasi, Ghana; 9 Department of (Experimental) Vascular Medicine, Amsterdam University Medical Centers, Amsterdam, The Netherlands; 10 Department of Internal Medicine, Section Nephrology, Amsterdam University Medical Centers, Amsterdam, The Netherlands; 11 Division of Endocrinology, Diabetes, and Metabolism, Johns Hopkins University School of Medicine, Baltimore, USA

**Keywords:** chronic kidney disease, liver biomarkers, fatty liver index, liver–kidney axis, MASLD, sub-Saharan Africa

## Abstract

**Background:**

The liver–kidney axis has been increasingly recognised as a potential pathway linking metabolic dysfunction to chronic kidney disease (CKD). However, longitudinal evidence from sub-Saharan African populations remains limited. We therefore aimed to prospectively investigate the association of baseline liver biomarkers with incident CKD and its components over six years in a transcontinental cohort of Ghanaians living in rural and urban Ghana and Amsterdam, the Netherlands.

**Methods:**

Using data from the prospective Research on Obesity and Diabetes among African Migrants cohort, we examined baseline liver biomarkers (gamma-glutamyl transferase (GGT), alanine aminotransferase (ALT), aspartate aminotransferase (AST) and the fatty liver index (FLI) in relation to incident CKD over approximately six years among Ghanaians in rural and urban Ghana and Amsterdam, The Netherlands. CKD was defined according to the Kidney Disease: Improving Global Outcomes 2021 criteria using race-free CKD-EPI equations. We used Poisson regression with robust standard errors to estimate adjusted incidence rate ratios (aIRRs) per standard deviation increases, adjusting for demographic, lifestyle, metabolic, and clinical factors. In fully adjusted models, we additionally accounted for longitudinal change (Δ) in liver biomarkers to isolate the association of baseline hepatic dysfunction with subsequent CKD risk.

**Results:**

Among 1,832 participants free of CKD at baseline (mean age of 45.9 years, 63% female), the incidence of CKD was 11%, the incidence of albuminuria was 9.7%, and the incidence of decreased estimated glomerular filtration rate (eGFR) was 2.3% over the follow-up period. Incident CKD was independently associated with higher baseline GGT (aIRR = 1.12; 95% confidence interval (CI) = 1.01–1.23), ALT (aIRR = 1.27; 95% CI = 1.04–1.50), and AST (aIRR = 1.20; 95% CI = 1.04–1.34). Associations were primarily driven by albuminuria, while we observed no significant associations for decreased eGFR. In exploratory analyses, elevated FLI was associated with incident CKD (aIRR = 1.92; 95% CI = 1.14–3.18 and albuminuria (aIRR = 2.15; 95% CI = 1.24–3.68), suggesting a broader metabolic-hepatic phenotype linked to early renal injury. Effect estimates remained materially consistent after inverse probability weighting to address differential follow-up.

**Conclusions:**

In our study, markers of liver cell injury were associated with increased risk of CKD over six years in the African population. Our findings support a potential link between hepatic metabolic dysfunction and early renal injury, highlighting the need for integrated cardiometabolic risk assessment in sub-Saharan Africa.

Chronic kidney disease (CKD) is an escalating global health concern, particularly in low- and middle-income regions such as sub-Saharan Africa (SSA). A 2014 systematic review reported CKD prevalence rates of 12% in urban areas and 17% in rural SSA [1]. Similarly, 2023 research in Uganda, South Africa, and Malawi shows national CKD prevalence ranging from 10% to 20% [2]. Furthermore, our recent research demonstrated a 12% CKD incidence over six years in both rural and urban Ghanaian populations. Not only is this CKD incidence elevated among Africans at home, but even among African migrants in Europe, with our same study reporting an 8% CKD incidence over six years in the migrant population [3].

Classical risk factors like unhealthy lifestyles, hypertension, diabetes, and genetic factors (*e.g.* *APOL1* gene variants) are key drivers of CKD burden worldwide and in African populations [2]. However, they do not fully explain the increased incidence and prevalence in this region, where traditional prevention strategies, such as screening for and managing hypertension and diabetes or improving access to healthcare, have not been fully effective in reducing the disease burden. A deeper understanding of the aetiology of CKD in this context is essential for informing more effective strategies for predicting, preventing, and treating the disease.

The liver–kidney axis plays a crucial role in individuals’ overall health [4] and may offer new insights into the escalating CKD burden in African populations. The liver and kidneys share a vital physiological relationship: the liver detoxifies, regulates hormones, and activates vitamin D [5–7], while the kidneys maintain electrolyte balance, blood pressure, and waste excretion [8–11]. Dysfunction in one organ can therefore lead to dysfunction in the other. For instance, metabolic dysfunction-associated steatotic liver disease (MASLD) affects approximately 25% of the European population [12,13] and has been consistently associated with an increased risk of CKD in populations of European descent [13].

While emerging studies on the liver–kidney axis have been conducted in European-descent populations [12,13], the factors contributing to liver dysfunction in African populations may differ significantly, leading to unique liver–kidney interactions. For example, higher rates of infectious diseases like hepatitis B could cause immune-related kidney damage [14]. Distinct dietary patterns, such as consumption of foods with aflatoxins like groundnuts, could induce hepatitis and liver cirrhosis with subsequent consequences for the kidneys [15]. The use of herbal medications poses a unique risk, as some contain compounds that are directly toxic to both the liver and the kidneys [16]. Furthermore, MASLD, which shares risk factors for albuminuria, is also increasingly common due to rapid urbanisation and rising obesity [17,18]. This complex interaction between factors necessitates a deeper understanding of liver–kidney interactions in African populations. To date, only one study in Ethiopia has examined associations between liver enzymes, such as alanine aminotransferase (ALT) and aspartate aminotransferase (AST), and kidney function [19]. However, this study was limited by its cross-sectional design and small sample size. Consequently, a significant knowledge gap remains regarding the influence of liver health on CKD development in SSA populations.

To address this gap, we aimed to investigate the prospective association between baseline liver biomarkers and incident CKD over six years among Ghanaian populations residing in rural and urban Ghana and Amsterdam, The Netherlands.

## METHODS

In this study, we examined markers of liver cell injury, *i.e.* gamma-glutamyl transferase (GGT), ALT, AST, and a metabolic-hepatic phenotype using the fatty liver index (FLI) in relation to incident CKD and its components – albuminuria and decreased estimated glomerular filtration rate (eGFR). We further explored potential effect modification by age, sex, education, and geographical location (rural and urban Ghana, or internationally among Ghanaian migrants in Amsterdam, The Netherlands).

### Study design and population

We used data from the transcontinental population-based Research on Obesity and Diabetes among African Migrants (RODAM) prospective cohort, which aims to examine complex gene–environmental interactions and their role in the development of cardio-metabolic diseases among African migrants and non-migrants. The cohort details have been published previously [20]. Briefly, 4,573 Ghanaian adults (≥18 years) were enrolled between 2012 and 2015 across rural and urban Ghana and Amsterdam, the Netherlands. Recruitment used the 2010 census enumeration areas in Ghana and the Amsterdam municipal register as sampling frames. Baseline data collection included questionnaires, anthropometry, and blood samples. Follow-up occurred from 2019–2021, with response rates of 63% (rural Ghana), 44% (urban Ghana), and 68% (Amsterdam). Ethical approval was granted by the Committee on Human Research, Ghana, and the Institutional Review Board, University of Amsterdam.

### Chronic kidney disease

CKD was defined using the Kidney Disease: Improving Global Outcomes (KDIGO) [21] criteria: an eGFR below 60 mL/min/1.73 m^2^ (stages 3a to 5) and/or ACR of ≥3 mg/mmol, with eGFR calculated using the 2021 race-free CKD-EPI equation [22]. For individuals with eGFR in the normal to mildly decreased range (stages 1 and 2), CKD was diagnosed using albuminuria (*i.e.* ACR ≥ 3 mg/mmol) as an additional diagnostic criterion, indicating kidney damage despite preserved kidney function. Therefore, individuals with albuminuria and a normal eGFR were considered to have CKD (Appendix S1 in the **Online Supplementary Document**).

### Biomarkers of liver cell damage

GGT, ALT, and AST were used as markers of liver cell damage. We measured GGT, ALT, and AST levels in heparin plasma samples from participants using a particle-enhanced immunoturbidimetric assay on the Pentra 400 Chemistry Analyzer (HORIBA ABX). The GGT levels were evaluated both as continuous and by World Health Organization cut-offs; as normal if below 55 U/L in males and 38 U/L in females; elevated if 55 U/L or higher in males and 38 U/L or higher in females. ALT and AST levels were also evaluated as continuous and also considered normal if below 40 U/L and elevated if above 40 U/L for both males and females [23].

### Exploratory analysis of FLI

In an exploratory secondary analysis using the FLI index, we examined whether FLI, which was developed in individuals aged 18–75 [24], was associated with CKD outcomes. The FLI was calculated using body mass index (BMI), waist circumference, triglycerides (TGs), and GGT based on the algorithm by Bedogni *et al.* [24], with scores ranging from 0 to 100. An FLI below 30 predicts the absence of MASLD with 91.5% sensitivity, while an FLI of 60 or higher predicts hepatic steatosis with 82.3% specificity. In the RODAM database, TGs were converted from mmol/L to mg/dL to match the FLI formula. Participants with duplicated IDs, those receiving antiretroviral therapy, those treated for hepatitis C, those with excessive alcohol use (over 21 units/168g per week for men, over 14 units/112g for women), and those older than 75 years were excluded from the FLI analysis [17].

### Other measurements

We retrieved the following measures from the RODAM cohort: age, sex, education levels, tobacco use, dietary patterns, alcohol consumption, anthropometrics, blood pressure, fasting blood glucose, lipids, hypertension, diabetes, and medication use (Appendix S1 in the **Online Supplementary Document**).

### Statistical analyses

For liver biomarkers, we presented baseline characteristics as means (x̄) and standard deviations (SDs) for normally distributed data, medians (Mdn) and interquartile ranges (IQRs) for skewed data, and frequencies and percentages for categorical data. As missing data was minimal (<5%) (Table S1 in the **Online Supplementary Document**), we used the unimputed dataset for analysis.

To reduce the likelihood that transient inflammatory or infectious states influenced baseline liver biomarkers, we excluded participants with symptoms of acute infection or current use of systemic anti-inflammatory, antibacterial, antifungal, or antiviral medications from the analysis. Because chronic infectious exposures (*e.g.* hepatitis B) and hepatotoxic environmental factors may not have been fully captured in the dataset, residual confounding by unmeasured liver-related conditions could not be excluded. We analysed continuous Z-standardised liver biomarkers as primary exposures to preserve statistical power and assess potential dose-response relationships. In addition, categorical analyses using established clinical cut-offs (elevated *vs.* non-elevated) were performed to facilitate clinical interpretability.

We used Poisson regression with robust errors to examine associations between GGT, ALT, and AST levels and CKD incidence after six years, and separately for albuminuria and decreased eGFR to explore differential outcome patterns. Given that exact time-to-event data were unavailable and CKD incidence was assessed at two study waves approximately six years apart, cumulative incidence rather than person-time incidence could be estimated. Robust Poisson regression was therefore used to directly estimate incidence rate ratios (IRRs), which approximate risk ratios and are more interpretable than odds ratios when outcomes are not rare. Models were built sequentially and included the crude model 1; model 2, which was adjusted for age and sex; model 3, which was further adjusted for education; and model 4, which was fully adjusted for demographic, lifestyle, anthropometric, metabolic and clinical variables, including BMI, obesity, diabetes, hypertension, physical activity, smoking, alcohol use, and relevant medication variables.

To account for potential changes in liver biomarker levels over time, fully adjusted models additionally included the change (Δ) in each respective liver biomarker between baseline and follow-up, thereby isolating the association of baseline levels with subsequent CKD risk independent of biomarker trajectories. In secondary longitudinal analyses, change (Δ) in liver biomarkers was also examined as the primary exposure to evaluate whether biomarker progression predicted CKD incidence. Given the limited power for detecting interaction effects and the increased risk of false-positive findings due to multiple testing, interaction analyses by age, sex, education, and geographical location were prespecified as exploratory (Appendix S1 in the **Online Supplementary Document**).

For the FLI analysis, baseline characteristics of participants with non-elevated FLI (<60) and elevated FLI (≥60) were presented similarly for categorical and continuous variables. Poisson regression with robust errors assessed associations between FLI scores and CKD incidence (also by albuminuria and decreased eGFR), adjusting for demographic, lifestyle, anthropometric, and medication factors. Interaction analyses examined variations by age, sex, education, and geographical location. Analyses were performed using *R*, version 4.2.2 (R Core Team, Vienna, Austria).

### Sensitivity analysis

To assess loss-to-follow-up bias, we compared baseline characteristics of included and excluded participants using *t*-tests for continuous variables and chi-squared tests for categorical variables. To further address potential selection bias due to differential follow-up, we performed inverse probability weighting (IPW). A logistic regression model was used to estimate each participant’s probability of having complete follow-up data based on baseline characteristics associated with follow-up status (age, sex, study site, baseline eGFR, albuminuria, hypertension, and diabetes). Stabilised inverse probability weights were derived from these predicted probabilities and applied to the fully adjusted Poisson regression models. Weighted effect estimates were compared with the primary analyses to evaluate the robustness of associations. The direction and magnitude of any attenuation or amplification of effect estimates following IPW were examined to assess the potential impact of differential follow-up on the primary findings. Additionally, we checked whether having more than one elevated liver marker was associated with an even greater risk of CKD.

## RESULTS

### Baseline characteristics of participants

**Figure 1:**
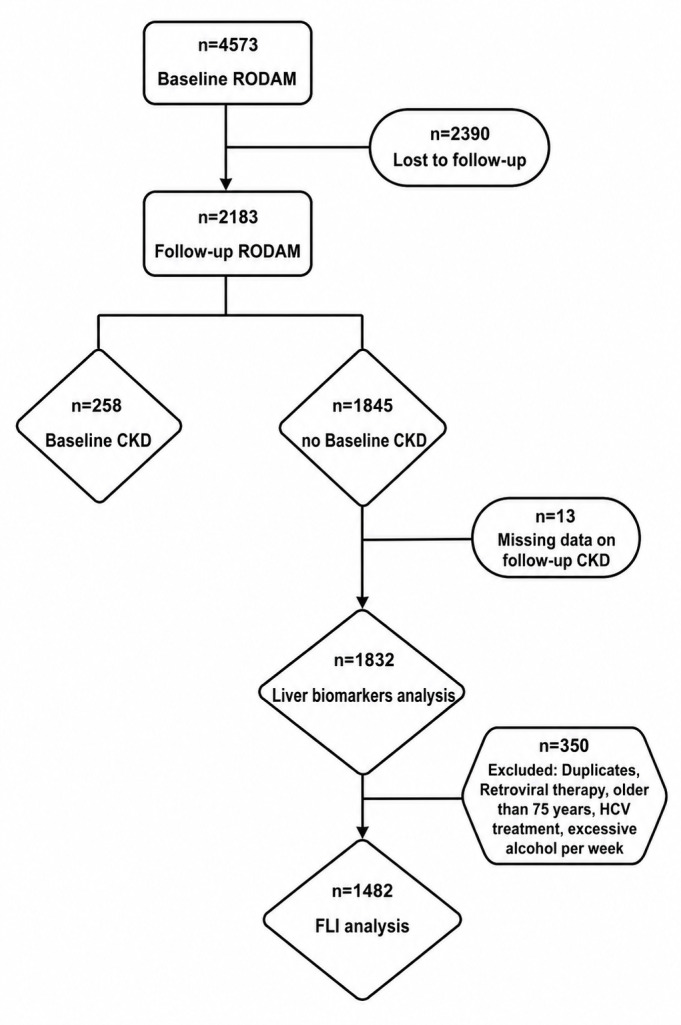
Flow diagram of participant inclusion and exclusion in the liver–kidney axis analysis.

Of the 4,573 eligible RODAM participants, 2,390 (52%) were lost to follow-up, leaving 2,183 participants. After excluding 13 with missing CKD outcome data, 1,832 participants were included in the liver biomarkers analysis. The FLI analysis included 1,482 participants free from anti-retroviral therapy, hepatitis C virus treatment, excessive alcohol consumption, and aged ≤75 years (**Figure 1**).

The study sample of 1,832 participants without CKD at baseline included 552 (30.1%) rural Ghanaians, 516 (28.2%) urban Ghanaians, and 764 (41.7%) Ghanaian migrants in Amsterdam. The mean age was 45.9 years, and 63.2% of participants were female. Most participants had lower vocational education (38.2%) and were employed part-time (45.9%). The median BMI was 26.0 kg/m^2^, with 23.1% obese. Alcohol consumption was reported by 38.2%, while the prevalence of smoking was low (2.7% current, 6.8% past). High physical activity was common (57.4%), albuminuria was present in 5.9%, hypertension in 38.8%, and diabetes in 6.4%, with 14.8% prescribed hypertension medication. Elevated liver enzymes were observed in 22.4% for GGT, 5.7% for ALT, and 22.4% for AST. CKD incidence was 10.9%, albuminuria incidence was 9.7%, and decreased eGFR was 2.3% (**Table 1**).

**Table 1:** Baseline characteristics of participants free of CKD at baseline (n = 1,832)

	Values
**Baseline characteristics, n (%)**	
Ghanaians in rural areas	552 (30.1)
Ghanaians in urban areas	516 (28.2)
Ghanaians in Amsterdam, The Netherlands	764 (41.7)
**Demographics**	
Age, x̄ (SD)	46 (11)
Sex, n (%)	
*Females*	1,157 (63.2)
Education, n (%)	
*Lower vocational*	655 (37.6)
*Intermediate*	281 (16.1)
*Higher vocational*	93 (5.3)
Employment status, n (%)	
*Full time*	561 (32.8)
*Part-time*	785 (45.9)
*Social benefits*	113 (6.6)
*Retired*	17 (1.0)
*Unable to work*	104 (6.1)
*Student*	20 (1.2)
**Anthropometry information, Mdn (IQR)**	
BMI (kg/m2)	26.0 (22.4-29.7)
Waist-hip ratio	0.90 (0.85-0.94)
**Lifestyle information**	
Any alcohol consumption, n (%)	578 (38.2)
Smoking, n (%)	
*Present*	47 (2.7)
*Past*	117 (6.8)
Physical activity, n (%)	
*Moderate*	258 (19.6)
*High*	754 (57.4)
**Laboratory information**	
Albuminuria, n (%)	108 (5.9)
Triglycerides (mmol/L), Mdn (IQR)	0.86 (0.64-1.16)
Cholesterol(mmol/L), Mdn (IQR)	4.84 (4.11-5.55)
Uric acid (µmol/L), Mdn (IQR)	294 (245-352)
Urine albumin(mg/L), Mdn (IQR))	2.0 (2.0-3.0)
Urine creatinine(mmol/L), Mdn (IQR)	10 (6-15)
Albumin creatinine ratio(mg/mmol), Mdn (IQR)	706.0 (375.0-1036.0)
eGFR, Mdn (IQR)	86 (76-100)
**Underlying conditions**	
Hypertension, n (%)	710 (38.8)
Diabetes, n (%)	117 (6.4)
Obesity, n (%)	423 (23.1)
**Use of medication for the underlying health conditions**	
Hypertension medication, n (%)	272 (14.8)
Diabetes medication, n (%)	48 (2.6)
**Biomarkers of liver cell damage**	
Gamma Glutamyl Transferase u/l, IQR, median (IQR)	
*Elevated, n (%)*	341 (22.4)
Alanine Transaminase u/l, median (IQR)	
*Elevated, n (%)*	87 (5.7)
Aspartate Transaminase u/l, median (IQR)	
*Elevated, n (%)*	341 (22.4)
**Follow-up CKD outcomes**	
CKD incidence, n (%)	
*Yes*	200 (10.9)
Albuminuria, n (%)	
*Yes*	164 (9.7)
eGFR	
*Decreased (<60), n (%)*	42 (2.3)

BMI – body mass index, eGFR – estimated glomerular filtration rate, IQR – interquartile range, Mdn – median, SD – standard deviation, x̄ – mean

### Association of biomarkers of liver damage with CKD outcomes: overall pattern across outcomes

Across all three liver enzymes, associations with incident CKD were predominantly driven by albuminuria rather than decreased eGFR, with no consistent associations observed for reduced eGFR (<60 mL/min/1.73 m^2^).

### Association of GGT with CKD markers at follow-up

A one SD increase in GGT was associated with a higher risk of incident CKD (adjusted IRR (aIRR) = 1.12; 95% CI = 1.01–1.23) (**Table 2**). GGT was also positively associated with albuminuria (aIRR = 1.27; 95% CI = 1.10–1.43), but not with decreased eGFR (aIRR = 0.99; 95% CI = 0.94–1.05).

**Table 2:** Association between baseline z-standardised liver biomarkers (GGT, ALT, AST) and incident CKD, albuminuria, and decreased eGFR over six years, presented as IRR (95% CI)*

Liver biomarkers	Model 1	Model 2	Model 3	Model 4
**CKD incidence (KDIGO criteria)**				
GGT	1.12 (1.02–1.19)	1.17 (1.06–1.25)	1.17 (1.06–1.26)	1.12 (1.01–1.23)
ALT	1.04 (0.91–1.13)	1.06 (0.94–1.12)	1.07 (0.96–1.15)	1.27 (1.04–1.50)
AST	1.08 (0.97–1.17)	1.10 (0.98–1.19)	1.11 (0.98–1.20)	1.20 (1.04–1.34)
**Albuminuria**				
GGT	1.20 (1.06–1.31)	1.29 (1.14–1.43)	1.28 (1.13–1.41)	1.27 (1.10–1.43)
ALT	1.03 (0.87–1.12)	1.05 (0.92–1.12)	1.07 (0.94–1.15)	1.26 (1.01–1.51)
AST	1.11 (0.98–1.21)	1.12 (1.01–1.21)	1.14 (1.01–1.24)	1.28 (1.08–1.49)
**eGFR**				
GGT	0.99 (0.94–1.04)	0.99 (0.94–1.04)	0.99 (0.94–1.04)	0.99 (0.94–1.05)
ALT	0.99 (0.94–1.04)	0.99 (0.94–1.04)	0.99 (0.93–1.04)	0.99 (0.90–1.07)
AST	0.99 (0.94–1.04)	1.01 (0.94–1.05)	1.01 (0.94–1.05)	0.99 (0.92–1.06)

ALT – alanine aminotransferase, AST – aspartate aminotransferase, CI – confidence interval, CKD – chronic kidney disease, eGFR – estimated glomerular filtration rate, GGT – gamma-glutamyl transferase, IRR – incidence rate ratio, KDIGO – Kidney Disease: Improving Global Outcomes

*There were 1832 participants in the analysis; 13 were excluded due to missing CKD parameters. Results based on robust Poisson regression. Predictor = GGT, ALT, or AST; outcome = CKD, albuminuria, or eGFR. CKD is defined based on the race-free CKD-EPI 2021 equation. Albuminuria is categorised as ACR <3 and ≥3. eGFR categorised as <60 and ≥60. Model 1 = unadjusted. Model 2 = adjusted for age and sex. Model 3 = model 2 + education. Model 4 = model 3 + smoking, physical activity, change (Δ) in biomarker (from baseline to follow-up), alcohol consumption, obesity, diabetes, uric acid, and hypertension.

Categorical analyses using established clinical cut-offs showed that elevated GGT was associated with a 45% higher risk of incident CKD (aIRR = 1.45; 95% CI = 1.02–2.03). For albuminuria, the association attenuated after adjustment (aIRR = 1.42; 95% CI = 0.97–2.04). No significant association was observed for decreased eGFR. Associations remained materially unchanged after additional adjustment for change (Δ) in GGT levels between baseline and follow-up. We identified significant interactions with age, female sex, and urban residence among Ghanaians and those who migrated to Amsterdam (Tables S2 and S3 in the **Online Supplementary Document**).

### Association of ALT with CKD markers at follow-up

A one SD increase in ALT was associated with a 27% higher risk of incident CKD (aIRR = 1.27; 95% CI = 1.04–1.50). ALT was also associated with albuminuria (aIRR = 1.26; 95% CI = 1.01–1.51), but not with decreased eGFR (aIRR = 0.99; 95% CI = 0.90–1.07) (**Table 2**).

Using clinical cut-offs, elevated ALT was associated with more than a two-fold increased risk of incident CKD (aIRR = 2.17; 95% CI = 1.17–3.70). No statistically significant associations were observed for albuminuria (aIRR = 1.77; 95% CI = 0.85–3.28) or decreased eGFR. Effect estimates were robust to additional adjustment for change (Δ) in ALT over time. We found no significant interactions with age, female sex, urban Ghanaians and those who migrated to Amsterdam (Tables S2 and S3 in the **Online Supplementary Document**)

### Association of AST with CKD markers at follow-up

A one SD increase in AST was associated with a 20% higher risk of incident CKD (aIRR = 1.20; 95% CI = 1.04–1.34). AST was also positively associated with albuminuria (aIRR = 1.28; 95% CI = 1.08–1.49), but not with decreased eGFR (aIRR = 0.99; 95% CI = 0.92–1.06) (**Table 2**).

Categorical analyses showed that elevated AST was associated with a 69% higher risk of CKD (aIRR = 1.69; 95% CI = 1.19–2.37) and an 80% higher risk of albuminuria (aIRR = 1.80; 95% CI = 1.24–2.58). No significant association was found with decreased eGFR. Associations persisted after accounting for longitudinal changes in AST levels. We found no significant interactions with age, female sex, urban Ghanaians and those who migrated to Amsterdam (Tables S2 and S3 in the **Online Supplementary Document**).

### Longitudinal change in liver biomarkers and CKD outcomes

To evaluate whether progression of liver injury predicted CKD risk beyond baseline levels, we examined longitudinal change (Δ, per SD increase) in each biomarker over six years (**Table 3**).

**Table 3:** Association between longitudinal change (Δ) in liver biomarkers and CKD outcomes over six years, presented as IRR (95% CI)*

Change in liver biomarkers	Model 1	Model 2	Model 3	Model 4
**CKD incidence (KDIGO criteria)**				
Δ GGT	1.10 (1.02–1.20)	1.12 (1.03–1.22)	1.11 (1.02–1.21)	1.09 (1.00–1.19)
Δ ALT	1.00 (0.86–1.16)	1.01 (0.86–1.19)	1.00 (0.84–1.19)	1.12 (0.95–1.32)
Δ AST	1.06 (0.97–1.16)	1.09 (0.99–1.18)	1.09 (1.00–1.18)	1.09 (1.02–1.17)
**Albuminuria**				
Δ GGT	1.12 (1.01–1.20)	1.13 (1.02–1.22)	1.12 (1.01–1.20)	1.13 (1.04–1.21)
Δ ALT	1.01 (0.88–1.15)	1.03 (0.89–1.20)	1.03 (0.88–1.20)	1.12 (0.92–1.29)
Δ AST	1.06 (0.97–1.16)	1.09 (0.99–1.19)	1.09 (1.00–1.19)	1.10 (1.02–1.18)
**eGFR**				
Δ GGT	0.99 (0.94–1.05)	0.99 (0.94–1.05)	0.99 (0.94–1.05)	0.99 (0.94–1.05)
Δ ALT	1.00 (0.95–1.05)	1.00 (0.95–1.05)	1.00 (0.95–1.06)	1.00 (0.93–1.07)
Δ AST	1.00 (0.95–1.05)	1.00 (0.95–1.05)	1.00 (0.95–1.05)	1.00 (0.94–1.06)

ALT – alanine aminotransferase, AST – aspartate aminotransferase, BMI – body mass index, CI – confidence interval, CKD – chronic kidney disease, eGFR – estimated glomerular filtration rate, GGT – gamma-glutamyl transferase, IRR – incidence rate ratio, KDIGO – Kidney Disease: Improving Global Outcomes, SD – standard deviation

*Results based on robust Poisson regression. Predictor = change (Δ) in GGT, ALT, or AST; outcome = CKD, albuminuria, or eGFR. CKD is defined based on the race-free CKD-EPI 2021 equation. Albuminuria is categorised as ACR <3 and ≥3. For decreased eGFR analysis, outcome was coded as 1 = eGFR ≥60 and 0 = eGFR <60; therefore, IRR < 1 indicates increased risk of reduced kidney function. Model 1 = unadjusted. Model 2 = adjusted for age and sex. Model 3 = adjusted for age, sex, and education. Model 4 = fully adjusted for age, sex, education, baseline biomarker level, BMI, obesity, diabetes, hypertension, physical activity, smoking, and alcohol use. Change (Δ) represents the difference between follow-up and baseline biomarker levels, standardised as a *z*-score. Estimates are per 1 SD increase in biomarker change.

For incident CKD, ΔGGT was positively associated with risk across models, with the fully adjusted IRR of 1.09 (95% CI = 1.00–1.19). ΔAST was similarly associated with CKD incidence (fully adjusted IRR = 1.09; 95% CI = 1.02–1.17). ΔALT was not significantly associated with CKD (fully adjusted IRR = 1.12; 95% CI = 0.95–1.32).

For albuminuria, similar patterns were observed. ΔGGT remained associated in the fully adjusted model (aIRR = 1.13; 95% CI = 1.04–1.21), and ΔAST showed consistent associations (aIRR = 1.10; 95% CI = 1.02–1.18). ΔALT was not significantly associated. In contrast, no significant associations were observed between change in liver biomarkers and decreased eGFR across any model (all IRRs approximately 1.00).

These findings indicate that progression in GGT and AST over time is associated with increased risk of CKD, particularly through incident albuminuria, whereas reduced eGFR was not significantly predicted by biomarker change.

### Exploratory analysis of FLI

Participants with elevated FLI differed significantly from those with non-elevated FLI in demographics, anthropometry, and clinical characteristics. For example, 73.1% of those with elevated FLI were obese, compared to 7.0% in the non-elevated group, and albuminuria prevalence was 9.0% *vs.* 5.1%. FLI was calculated for 1,482 participants, with an average score of 36 (Table S4 in the **Online Supplementary Document**).

When analysed as a continuous variable (per unit increase), FLI was not significantly associated with incident CKD in fully adjusted models (IRR = 1.01; 95% CI = 0.99–1.01). For albuminuria, however, higher FLI was modestly associated with increased risk in the fully adjusted model (IRR = 1.02; 95% CI = 1.01–1.03). No significant associations were observed between continuous FLI and decreased eGFR across any model (all IRRs approximately 1.00) (Table S5 in the **Online Supplementary Document**).

When categorised using the established clinical cut-off (FLI ≥ 60), elevated FLI was associated with a substantially higher risk of incident CKD in the fully adjusted model (IRR = 1.92; 95% CI = 1.14–3.18). Elevated FLI was also associated with more than a two-fold increased risk of albuminuria (aIRR = 2.15; 95% CI = 1.24–3.68). No association was observed for decreased eGFR (aIRR = 1.01; 95% CI = 0.82–1.21). Interactions with age, sex, and geographical location were not significant. The 346 excluded participants were predominantly male (58.7%) and Ghanaian migrants in Amsterdam (95.1%), with higher rates of hypertension (49.4%) and obesity (28.4%) (Tables S5–7 in the **Online Supplementary Document**).

### Sensitivity analysis

The sensitivity analysis revealed significant differences between participants included in the study and those lost to follow-up. Rural Ghanaians were more likely to be included, while urban and Amsterdam residents were more likely to be lost (*P* < 0.001). Those lost to follow-up had higher rates of albuminuria (14.2% *vs.* 5.9%, *P* < 0.001), hypertension (44.9% *vs.* 38.8%, *P* < 0.001), and diabetes (9.7% *vs.* 6.4%, *P* < 0.001), as well as slightly elevated ALT (*P* = 0.026) and AST levels (*P* < 0.001), indicating a higher liver damage burden. No significant differences were observed in GGT levels or metabolic profiles like BMI, eGFR, or triglycerides. Importantly, participants lost to follow-up exhibited a higher baseline risk profile, suggesting that attrition may have biased associations toward the null rather than inflated effect estimates (Table S8 in the **Online Supplementary Document**).

Inverse probability weighted analyses yielded effect estimates that were highly comparable to the fully adjusted primary models. The magnitude and direction of associations remained materially unchanged for CKD incidence, albuminuria, and decreased eGFR after accounting for differential follow-up. For example, the fully adjusted IRR for GGT and CKD incidence was 1.12 (95% CI = 1.01–1.23) in the primary analysis and 1.15 (95% CI = 1.03–1.26) after IPW. Similar consistency was observed for ALT and AST. These findings indicate that after accounting for differential follow-up through IPW, the associations remained robust in both direction and magnitude. This reduces the likelihood that selection bias meaningfully influenced the primary conclusions. Analysis of liver marker combinations showed that having two or more elevated markers was linked to significantly higher CKD risk, albuminuria, and decreased eGFR over six years (Tables S9 and S10 in the **Online Supplementary Document**).

## DISCUSSION

We explored the liver–kidney axis in an African population by investigating associations between liver pathology markers (GGT, ALT, AST, and FLI) and CKD incidence six years later among Ghanaians. Our findings indicate that elevated liver enzymes (GGT, ALT, and AST) and high FLI scores are positively associated with CKD incidence. Importantly, these associations were predominantly driven by albuminuria rather than reduced eGFR, suggesting a relationship with early renal injury rather than advanced loss of filtration capacity. Associations were observed using continuous Z-standardised biomarkers and remained robust after accounting for longitudinal changes in liver enzyme levels. In longitudinal analyses, increases in GGT and AST over time were also associated with higher CKD risk, reinforcing the potential relevance of dynamic hepatic dysfunction. Together, these findings support a potential link between hepatic metabolic disturbance and early kidney damage in this population.

We found a positive association between elevated GGT levels and CKD incidence six years later, which aligns with findings from studies in European, Asian, and USA populations where elevated GGT levels have been linked to the prevalence or incidence of CKD [23,25,26]. Notably, GGT plays a central role in glutathione metabolism and is considered a marker of oxidative stress and systemic inflammation [23]. Beyond its role as a hepatic enzyme, circulating GGT has been proposed as an integrative marker of redox imbalance, which may contribute to endothelial dysfunction and glomerular injury. In the African context, elevated GGT levels may be influenced by multiple factors, including environmental toxins and infections, which increase oxidative stress in the liver. However, alcohol consumption, which is a well-known cause of elevated GGT levels, was not an explaining factor in our cohort. This is important as alcohol can directly raise GGT levels. Although we did not measure environmental toxins or infections in our study, other research from SSA has consistently shown higher GGT levels under these conditions [27–29]. For example, studies from Ghana have shown that GGT levels can be two times higher in individuals with malaria compared to those without the disease [30], which has a 40% annual prevalence in the country [31]. This suggests that malaria may contribute to increased oxidative damage in the liver and potentially intensify liver–kidney interactions in this context. Other research from Africa has also demonstrated higher GGT levels in conditions like hepatitis B and C and HIV [27,28]. Furthermore, we observed no significant association between elevated GGT levels and decreased eGFR in our cohort, but did identify an association with albuminuria. This highlights the potential role of GGT in early renal dysfunction, which may not yet manifest in reduced eGFR but could be detected through elevated urine albumin levels.

While liver enzymes may fluctuate due to transient infections or environmental exposures common in SSA, several methodological features strengthen interpretation. Participants with acute infections or inflammatory medication use were excluded at baseline, and fully adjusted models accounted for change (Δ) in liver biomarkers over time. Furthermore, longitudinal analyses demonstrated that increases in GGT over follow-up were independently associated with CKD and albuminuria risk, suggesting that worsening hepatic injury may parallel renal vulnerability. Although chronic hepatic dysfunction cannot be definitively distinguished from transient elevations, the consistency across baseline and longitudinal models supports a more sustained underlying metabolic or inflammatory process.

The association between elevated GGT and CKD was more pronounced in women, older participants, and those in urban Ghana and Amsterdam settings. These differences should be interpreted cautiously, as interaction analyses were exploratory; however, they may reflect differential metabolic vulnerability across demographic and migration contexts. The stronger association in females may be linked to hormonal differences, particularly the protective effect of oestrogen on both liver and kidney function [32]. Postmenopausal women may experience a loss of this protection, increasing their susceptibility to oxidative stress and metabolic disorders, which could explain their heightened risk for CKD in this cohort. Age is another key factor, as older individuals have reduced antioxidant defences and are more likely to accumulate metabolic and inflammatory damage over time, exacerbating both liver and kidney dysfunction [33]. Urbanisation within SSA and migration to higher-income countries, such as The Netherlands, are linked to higher metabolic risk profiles, including obesity, hypertension, and poor dietary habits, which increase the likelihood of both liver and kidney disease [34]. These factors were adjusted for in our analysis to isolate the independent effects of liver biomarkers and FLI on CKD incidence. These findings support the potential value of targeting metabolic risk factors in urban and migrant populations.

ALT and AST, traditional markers of liver function, were significantly associated with CKD in our study, consistent with findings from Japan and Europe [35,36]. ALT and AST elevations can be due to a range of liver conditions, including viral hepatitis (B and C), autoimmune hepatitis, cirrhosis and MASLD [29,37]. Medications such as statins, acetaminophen, and certain antibiotics are also known to elevate ALT and AST levels [38,39]. While we included lipid-lowering medications, primarily statins, in our study data, we lacked detailed information on other exposures, such as over-the-counter medications, limiting our ability to fully assess their contributions to elevated ALT levels. However, previous research in African populations has documented higher ALT and AST levels in viral hepatitis [14,40]. Across various regions in Africa, the prevalence of viral hepatitis ranges from 10.9% to 38% [41,42], and these infections may also contribute to elevated ALT levels in our study population.

Additionally, the overuse of antibiotics in Africa is well-documented, with studies showing that antibiotic use can lead to liver enzyme elevation, including AST and ALT [43,44]. In the SSA region, up to 93.9% of the population may use antibiotics without a prescription [45], further compounding the risk of liver damage. Furthermore, herbal medication use is prevalent in many African countries, with individuals relying on traditional remedies for diseases like hypertension [46], some of which have been linked to liver toxicity and elevated AST and ALT levels [47,48]. These unique factors could partially explain the elevated liver enzyme levels observed in our study. Furthermore, we found no significant association between elevated ALT levels and eGFR, suggesting that ALT may not have a direct impact on glomerular filtration in the early stages of CKD. However, a positive association between elevated ALT and albuminuria indicates that ALT may be involved in early renal damage, which may manifest, since we observed increased albumin leakage in the urine before eGFR decline. Similarly, elevated AST levels were positively associated with albuminuria, reinforcing the possibility that liver dysfunction may contribute to early renal injury, especially in populations at higher risk for oxidative stress and liver–kidney interactions. Our longitudinal change analyses provide additional insight. Increases in GGT and AST over time were associated with higher CKD risk even after adjustment for baseline levels and cardiometabolic factors, whereas change in ALT was not consistently associated. These findings suggest that dynamic hepatic deterioration may carry prognostic information beyond baseline categorisation, although further replication is warranted.

In exploratory analyses, elevated FLI, a surrogate marker of MASLD, was associated with nearly twofold higher CKD and albuminuria risk. When analysed continuously, however, FLI showed only modest associations, indicating that clinically significant hepatic-metabolic burden rather than incremental variation may drive risk. Our findings are consistent with those in European and Asian populations where MASLD has been increasingly examined for its potential role as a CKD risk factor [49,50]. The prevalence of MASLD in SSA is increasing, driven by urbanisation and lifestyle changes that have led to higher rates of obesity, diabetes, and hypertension. For instance, recent studies estimate the prevalence of MAFLD in SSA ranges from 10% to 40%, depending on the population studied [51]. Physiologically, the liver’s role in glucose and lipid metabolism suggests that liver fat accumulation in MASLD may exacerbate insulin resistance, dyslipidaemia, and chronic inflammation, pathways contributing to kidney damage in other settings [52]. Emerging evidence highlights immune-mediated mechanisms in the liver–kidney axis, including low-grade inflammation, subclinical portal hypertension, and the hepato-renal reflex, which are areas of ongoing research. For instance, liver inflammation has been independently associated with an increased risk of CKD and ESRD in prospective studies [53]. While our findings are consistent with these mechanistic hypotheses, FLI is a composite index that incorporates anthropometric and metabolic components that are themselves associated with CKD risk. Therefore, FLI in this context likely reflects a broader metabolic–hepatic phenotype rather than isolated hepatic steatosis. Moreover, FLI was originally validated in European populations, and region-specific validation in SSA cohorts remains limited. Interventions targeting metabolic risk factors, including glucagon-like peptide receptor agonists such as semaglutide, have shown promise, with studies indicating they can halve albuminuria in obese individuals, offering dual benefits for MASLD and CKD risk management [54]. Furthermore, imaging-based validation of hepatic steatosis was not available in our study, and region-specific validation of FLI against ultrasound or elastography in SSA populations remains limited. Consequently, these findings should be interpreted cautiously and considered hypothesis-generating. Further studies incorporating direct imaging measures of liver fat are needed to clarify the independent contribution of hepatic steatosis to CKD risk in African populations.

Our results have important public health and clinical implications. The findings demonstrate that markers of liver cell injury (GGT, ALT, and AST) and a broader metabolic-hepatic phenotype, reflected by FLI, were associated with higher CKD risk over six years. These associations were primarily driven by albuminuria, suggesting that liver-related metabolic dysfunction may be linked to early renal injury. While causality cannot be established, our findings suggest that liver biomarkers may serve as early indicators of heightened CKD risk in African populations. Given the rising burden of metabolic disease in SSA, integrated liver–kidney risk assessment may help identify high-risk individuals who could benefit from targeted cardiometabolic prevention strategies. Importantly, although our fully adjusted models accounted for established metabolic risk factors including obesity, hypertension, diabetes, uric acid, and lifestyle behaviours, the associations between liver enzymes and CKD outcomes remained materially consistent. This suggests that traditional cardiometabolic pathways do not entirely explain the observed relationships. However, metabolic dysfunction likely represents a partial intermediary mechanism within the liver–kidney axis. Hepatic steatosis and hepatocellular injury may promote insulin resistance, dyslipidaemia, endothelial dysfunction, and chronic low-grade inflammation, which in turn contribute to early renal injury. Because metabolic factors can act both as confounders and potential mediators, formal causal mediation analysis would require repeated intermediate measurements and clear temporal separation between exposure, mediator progression, and outcome development. Such analyses were beyond the scope of the present study and may have been statistically underpowered given the number of CKD events. Future longitudinal studies incorporating repeated metabolic assessments, biomarker measurements, imaging-based liver assessment, and formal mediation modelling are needed to clarify the pathways linking hepatic dysfunction and CKD risk, including the extent to which these associations are driven by direct hepatic effects *vs.* metabolically mediated pathways.

### Strengths and limitations

Our study’s strengths include its longitudinal design, which allowed us to assess the association between liver biomarkers and CKD incidence over six years, providing insights into the long-term effects of these markers, compared to cross-sectional studies. The inclusion of participants from rural and urban Ghana, and Ghanaian migrants in Amsterdam enhances the diversity and generalizability of our findings, capturing a range of demographic and geographic contexts. We evaluated multiple liver biomarkers (GGT, ALT, AST, and FLI), providing a comprehensive analysis of both metabolic and non-metabolic liver function, and adjusted for important confounders such as age, sex, education, and lifestyle factors. Importantly, we examined both baseline biomarker levels and longitudinal change (Δ) over time, allowing us to differentiate static hepatic status from dynamic biomarker trajectories. Additionally, fully adjusted models accounted for baseline cardiometabolic factors and longitudinal change in liver biomarker levels, strengthening inference regarding the independent association of hepatic dysfunction with subsequent CKD risk. The consistency of findings across continuous, categorical, and longitudinal models further enhances internal validity.

However, several limitations of our study warrant consideration. There were significant differences in response and participation rates across study sites, with particularly lower follow-up rates in urban Ghana and among Ghanaian migrants in Amsterdam. This was largely due to the COVID-19 pandemic, which began midway through follow-up data collection. Lockdowns and fear of infection, especially in urban Ghana, where follow-up occurred during the pandemic, likely contributed to attrition. Participants lost to follow-up had higher baseline prevalences of albuminuria, hypertension, diabetes, and mildly elevated liver enzymes, indicating non-random attrition. This pattern suggests that higher-risk individuals were more likely to be lost, raising the possibility of selection bias. To address this concern, we conducted IPW analyses using baseline predictors of follow-up participation. The weighted effect estimates were materially consistent with the primary analyses, suggesting that differential loss to follow-up did not substantially alter the magnitude or direction of associations. Nevertheless, residual selection bias cannot be fully excluded, and effect estimates should be interpreted cautiously. Notably, attrition of higher-risk individuals would most plausibly bias associations toward the null, potentially resulting in conservative rather than inflated estimates.

Liver biomarkers were primarily assessed at baseline, and these markers may be influenced by transient conditions such as infections, medication use, or short-term environmental exposures. Although individuals with acute infections or inflammatory medication use were excluded at baseline, and models were adjusted for longitudinal change (Δ) in liver enzyme levels, we cannot fully exclude residual confounding due to unmeasured infectious diseases (*e.g.* hepatitis B or C), environmental hepatotoxins, or traditional/herbal medication use. Therefore, associations observed may reflect both metabolic and context-specific hepatic stressors. Repeated exposure measurements and more detailed assessment of infectious and hepatotoxic exposures would strengthen causal inference.

The use of FLI as a surrogate marker of hepatic steatosis represents an additional limitation. FLI incorporates metabolic parameters that are themselves associated with CKD risk, potentially introducing collinearity and limiting interpretation of liver-specific effects. Furthermore, FLI was originally validated in predominantly European populations, and validation against imaging-based measures of hepatic steatosis in SSA populations remains limited. Therefore, FLI findings should be interpreted as reflecting a broader metabolic-hepatic phenotype rather than isolated liver fat accumulation and should be considered exploratory.

A further limitation relates to the assessment of albuminuria at a single follow-up time point without confirmation of persistence over ≥3 months, as recommended by KDIGO guidelines [21]. In large population-based cohort studies, repeat short-interval measurements are often not feasible. Consequently, some cases classified as incident CKD may reflect transient albuminuria due to infection, dehydration, or physical exertion. Such misclassification would most likely be non-differential and bias associations toward the null rather than generate spurious positive findings. Given that associations were predominantly driven by albuminuria rather than decreased eGFR, our findings should be interpreted as reflecting incident renal impairment or albuminuria at follow-up rather than definitively confirmed chronic kidney disease.

The absence of data on herbal medicine use, *APOL1* genetic variants, and infectious diseases (*e.g.* hepatitis B/C, tuberculosis), which are highly relevant in SSA, limits our ability to fully account for CKD risk. These factors may contribute to both liver enzyme elevation and kidney dysfunction and could partly explain observed associations. Future studies incorporating genetic, infectious, and environmental exposure data are needed to disentangle metabolic from non-metabolic pathways within the liver–kidney axis in African populations**.** Additionally, kidney function was estimated using creatinine-based eGFR equations. Although we applied the 2021 race-free CKD-EPI equation, the absence of cystatin C or measured GFR methods such as iohexol clearance is noteworthy. These approaches are increasingly recommended for both African populations and the general population due to improved accuracy. If creatinine-based equations underestimate CKD risk, as suggested in some studies, the true burden of CKD in this population may be even higher than reported. This possibility is particularly concerning and further underscores the urgency of strengthening CKD detection, prevention, and surveillance efforts in SSA. Finally, while liver enzymes provide clinically accessible markers of hepatocellular injury, they are non-specific. Future research incorporating imaging-based liver assessment and inflammatory or fibrosis markers would further clarify the mechanistic pathways linking hepatic dysfunction to CKD risk.

## CONCLUSIONS

Our findings show that biomarkers of liver cell injury and a metabolic-hepatic phenotype, reflected by the FLI, were associated with increased risk of renal impairment, predominantly albuminuria, in populations in urban and rural Ghana and Amsterdam, The Netherlands, over six years. These findings support a role for the liver–kidney axis in early renal injury and highlight the potential value of integrated liver–kidney risk assessment in SSA.

## Additional material


Online Supplementary Document

## Data Availability

**Data availability:** The data that support the findings of this study are available from the corresponding author upon reasonable request. Due to privacy and ethical considerations, the data are not publicly available. Requests for access to the data should be directed to the corresponding author and will be considered by the RODAM study’s ethics committee. The data are stored in a secure database and can be accessed under the conditions set by the ethical guidelines of the RODAM study and the participating institutions.
