# Supplementary material for: Exploring the liver–kidney axis in the Ghanaian population: the RODAM prospective study
Source: J Glob Health. 2026 Jul 24;16:04252. doi: 10.7189/jogh.16.04252 (PMC13397099; doi:10.7189/jogh.16.04252)
Supplement: Online Supplementary Document [file jogh-16-04252-s001.pdf]

Supplement to: Mungamba MM, Chilunga FP, van der Linden EL, Henneman P, Hayfron-Benjamin CF, Meeks KAC, Darko SN, Twumasi-Ankrah S, Owusu-Dabo E, Holleboom AG, Vogt L, van den Born BJ, Nkeh-Chungag BN, Agyemang C. Exploring the liver-kidney axis in the Ghanaian population: the RODAM prospective study. J Glob Health. 2026;16:04252.

__Contents__

Supplementary Methods
Appendix 1. Detailed description of covariate assessment and definitions.

__Supplementary Tables__

Supplementary Table S1: Imputed characteristics of all participants
Supplementary Table S2: Association of liver markers categorized with CKD outcomes six years later.
Supplementary Table S3: Interaction between baseline liver biomarkers and demographic factors in the relationship with CKD
Supplementary Table S4: Baseline characteristics of non-elevated vs elevated FLI participants
Supplementary Table S5: Exploratory association between FLI and FLI categorized with CKD outcomes six years later
Supplementary Table S6: Interaction between baseline FLI and demographic factors in the relationship with CKD
Supplementary Table S7: Characteristics of all excluded participants
Supplementary Table S8: Baseline characteristics of included vs lost to follow-up
Supplementary Table S9: Comparison of fully adjusted associations between baseline z-standardised liver biomarkers and CKD outcomes with and without inverse probability weighting (IPW).
Supplementary Table S10: Association of liver- biomarkers composition with CKD incidence, albuminuria and decreased eGFR six years later.

Appendix 1: Detailed account of other measurements

Additional measurements

In addition to measurements related to chronic kidney disease (CKD) and liver biomarkers, we collected a comprehensive set of measurements from the RODAM cohort for analysis. These include age, sex, education levels, tobacco smoking, dietary patterns, alcohol consumption, physical activity, anthropometrics, blood pressure, fasting blood glucose, hypertension, diabetes, and use of medications

Age was recorded in years; and sex was categorized into male and female. Educational level was assessed based on the highest education attained, with categories including never been to school, lower vocational, intermediate vocational, and higher vocational. Smoking status was categorized as yes, no, or ex-smoker, while alcohol use in the last 12 months was categorized as yes or no. The WHO steps questionnaire was used to derive physical activity in metabolic equivalent (MET, hours/week), which included physical activity at work, while commuting, and in leisure time. Answers were subsequently classified based on the guidelines of The IPAQ group into three levels of total physical activity (low, moderate, high) (1-3).

Body weight was measured in light clothing without shoes, using SECA 877 scales with a precision of 0.1 kg. Height was measured without shoes using a portable stadiometer (SECA 217) with a precision of 0.1 cm. Body mass index (BMI) was calculated as weight divided by height squared (kg/m2). Overweight was defined as a BMI of between 25 and 30 kg/m2, while obesity was defined as a BMI of 30 kg/m2 or higher. Waist circumference was measured in centimeters at the midpoint between the lower rib and the upper margin of the iliac crest. All these anthropometric measures were taken twice, and the mean was used for analysis.

Blood pressure (BP) was measured three times while participants were in a seated position after resting for at least 5 minutes. The measurements were taken using the Microlife WatchBP Home, a validated semi-automated device. The mean of the last two BP measurements was used for analysis. Hypertension was defined as systolic BP of 140 mmHg or higher and/or diastolic BP of 90 mmHg or higher, or the use of antihypertensive medication per WHO criteria (2).

Fasting plasma glucose concentration was determined using the enzymatic hexokinase method. Type 2 diabetes was defined based on the diagnostic criteria set by the WHO, including a fasting glucose level of 7.0 mmol/L or higher, current use of medication prescribed for diabetes treatment, or self-reported physician-diagnosed diabetes (4, 5). Serum uric acid concentration was measured using an enzymatic method (Trinder) in µmol/L. For total cholesterol (TC), a ready to use reagent for colorimetry was used.

Medication use was assessed by asking participants to bring their prescribed medications to the physical exam and these were subsequently coded and categorized. For hypertension, antihypertensive medications included were diuretics, beta blockers, angiotensin converting enzyme (ACE) inhibitors, or

Angiotensin Receptor Blockers (ARBs), either individually or in combination. For diabetes, hypoglycemic medications were considered including Metformin, Sulfonylureas, and Insulin.

Chronic kidney disease

Participants provided early morning urine samples to analyse albumin and creatinine. Urinary-albumin-concentration (in mg/L) was determined using an immunochemical-turbidimetric-method and urinary-creatinine-concentration (in mmol/L) using kinetic spectrophotometric method (Roche Diagnostics). Albuminuria categories were based on urine albumin-to-creatinine ratio (ACR): A1 (<3 mg/mmol; normal to mildly increased), A2 (3-30 mg/mmol; moderately increased) and A3 (>30 mg/mmol; severely increased)(6).

Estimated glomerular filtration rate (eGFR) was calculated using the race-free CKD-EPI 2021 equation (7), selected for its improved accuracy in African populations and due to the absence of Iohexol-based measurements (8). Serum creatinine concentration (mmol/L) was determined using a kinetic colorimetric spectrophotometric isotope dilution mass spectrometry calibrated method (Roche Diagnostics).

CKD was defined using KDIGO criteria : an eGFR below 60 mL/min/1.73 m^2 (stages 3a to 5) and/or ACR of ≥3 mg/mmol (9). For individuals with eGFR in the normal to mildly decreased range (stages 1 and 2), CKD was diagnosed using albuminuria (i.e., ACR ≥ 3 mg/mmol) as an additional diagnostic criterion, indicating kidney damage despite preserved kidney function. Therefore, individuals with albuminuria and a normal eGFR were considered to have CKD.

References

1. Armstrong T, Bull F. Development of the world health organization global physical activity questionnaire (GPAQ). Journal of Public Health. 2006;14:66-70.
2. Organization WH. Guideline for the pharmacological treatment of hypertension in adults: World Health Organization; 2021.
3. Osei TB, van Dijk AM, Dingerink S, Chilunga FP, Beune E, Meeks KAC, et al. Reduced Rank Regression-Derived Dietary Patterns Related to the Fatty Liver Index and Associations with Type 2 Diabetes Mellitus among Ghanaian Populations under Transition: The RODAM Study. Nutrients. 2021;13(11).
4. Roth GA, Abate D, Abate KH, Abay SM, Abbafati C, Abbasi N, et al. Global, regional, and national age-sex-specific mortality for 282 causes of death in 195 countries and territories, 1980-2017: a systematic analysis for the Global Burden of Disease Study 2017. The lancet. 2018;392(10159):1736-88.
5. Committee ADAPP, Committee: ADAPP. 2. Classification and diagnosis of diabetes: Standards of Medical Care in Diabetes—2022. Diabetes care. 2022;45(Supplement_1):S17-S38.
6. Lameire NH, Levin A, Kellum JA, Cheung M, Jadoul M, Winkelmayer WC, et al. Harmonizing acute and chronic kidney disease definition and classification: report of a Kidney Disease: Improving Global Outcomes (KDIGO) Consensus Conference. Kidney international. 2021;100(3):516-26.
7. Miller WG, Kaufman HW, Levey AS, Straseski JA, Wilhelms KW, Yu HY, et al. National Kidney Foundation Laboratory Engagement Working Group recommendations for implementing the CKD-EPI 2021 race-free equations for estimated glomerular filtration rate: practical guidance for clinical laboratories. Clinical chemistry. 2022;68(4):511-20.
8. Fabian J, Kalyesubula R, Mkandawire J, Hansen CH, Nitsch D, Musenge E, et al. Measurement of kidney function in Malawi, South Africa, and Uganda: a multicentre cohort study. The Lancet Global Health. 2022;10(8):e1159-e69.
9. Levey AS, Eckardt K-U, Dorman NM, Christiansen SL, Hoorn EJ, Ingelfinger JR, et al. Nomenclature for kidney function and disease: report of a Kidney Disease: Improving Global Outcomes (KDIGO) Consensus Conference. Kidney international. 2020;97(6):1117-29.

Supplementary Table S1: Imputed characteristics of all participants

Variables	N or Mean or Median	% or SD or IQR
Baseline Characteristics		
Total numbers enrolled	1832	
Ghanaians in Rural, n (%)	552	30.1
Ghanaians in Urban, n (%)	516	28.2
Ghanaians in Amsterdam, n (%)	764	41.7
Demographics		
**Age, mean (SD)**	46	11
**Sex, n (%)**		
Females	1,157	63.2
**Education, n (%)**		
Lower vocational	666	38.2
Intermediate	303	16.5
Higher vocational	95	5.2
**Employment status, n (%)**		
Full time	564	32.6
Part-time	794	45.9
Social benefits	113	6.5
Retired	17	1.0
Unable to work	107	6.2
Student	20	1.2
Anthropometry information		
**BMI (kg/m2), median (IQR)**	26.0	22.4-29.7
**Waist hip ratio, median (IQR)**	0.90	0.85-0.94
Lifestyle information		
**Any alcohol consumption, n (%)**	698	38.1
**Smoking, n (%)**		
Present	47	2.6
Past	138	7.5
**Physical activity, n (%)**		
Moderate	317	17.3
High	956	52.2
Laboratory information		
**Albuminuria, n (%)**	108	5.9
**Triglycerides (mmol/L), median (IQR)**	0.86	0.64-1.16
**Cholesterol(mmol/L), median (IQR)**	4.84	4.11-5.55
**Uric acid (µmol/L), median (IQR)**	296	246-353
**Urine albumin(mg/L), median (IQR)**	2.0	2.0-3.0
**Urine creatinine(mmol/L), median (IQR)**	10	6-15
**Albumin creatinine ratio(mg/mmol), median (IQR)**	697	368-1023
**eGFR, median (IQR)**	86	76-100
Underlying conditions		
**Hypertension, n (%)**	710	38.8
**Diabetes, n (%)**	117	6.4
**Obesity, n (%)**	423	23.1
Use of medication for the underlying health conditions		
**Hypertension medication, n (%)**	272	14.8
**Diabetes medication, n (%)**	48	2.6
Biomarkers of Liver cell damage		
**Gamma Glutamyl Transferase u/l, IQR, median (IQR)**		
Elevated	423	23.1
**Alanine Transaminase u/l, median (IQR)**		
Elevated	91	5.0
**Aspartate Transaminase u/l, median (IQR)**		
Elevated	386	21.1
Follow-up CKD outcomes		
**CKD incidence, n (%)**		
Yes	200	10.9
**Albuminuria, n (%)**		
Yes	164	9.0
**eGFR**		
Decreased (<60)	42	2.3

Data are presented as percentages, means (SDs), or median (interquartile range). Percentages are rounded to one decimal point and may not sum to 100%. % = Percentages. eGFR= Estimated glomerular filtration rate. BMI=body mass index.

Supplementary Table S2: Association of liver markers categorized with CKD outcomes six years later.

Biomarkers	N	Model 1<br>IRR (95% CI)	Model 2<br>IRR (95% CI)	Model 3<br>IRR (95% CI)	Model 4<br>IRR (95% CI)
**GGT levels**					
CKD incidence (KDIGO criteria)					
GGT non-elevated	1181	1.00 (Reference)	1.00 (Reference)	1.00 (Reference)	1.00 (Reference)
GGT elevated	341	**1.68 (1.22-2.30)**	**1.60 (1.15-2.18)**	**1.59 (1.14-2.18)**	**1.45 (1.02-2.03)**
Albuminuria (outcome)					
GGT non-elevated	1524	1.00 (Reference)	1.00 (Reference)	1.00 (Reference)	1.00 (Reference)
GGT elevated	164	**1.67 (1.17-2.34)**	**1.60 (1.12-2.24)**	**1.57 (1.10-2.21)**	1.42 (0.97-2.04)
eGFR					
GGT non-elevated	990	1.00 (Reference)	1.00 (Reference)	1.00 (Reference)	1.00 (Reference)
GGT elevated	532	1.01 (0.90-1.14)	1.01 (0.89-1.13)	1.01 (0.89-1.14)	1.01 (0.88-1.15)
ALT levels					
CKD incidence (KDIGO criteria)					
ALT non-elevated	1435	1.00 (Reference)	1.00 (Reference)	1.00 (Reference)	1.00 (Reference)
ALT elevated	87	1.55 (0.87-2.55)	**2.05 (1.14-3.41)**	**2.23 (1.24-3.72)**	**2.17 (1.17-3.70)**
Albuminuria					
ALT non-elevated	1435	1.00 (Reference)	1.00 (Reference)	1.00 (Reference)	1.00 (Reference)
ALT elevated	87	1.29 (0.66-2.28)	1.77 (0.89-3.18)	1.90 (0.95-3.41)	1.77 (0.85-3.28)
eGFR					
ALT non-elevated	1435	1.00 (Reference)	1.00 (Reference)	1.00 (Reference)	1.00 (Reference)
ALT elevated	87	1.02 (0.82-1.26)	1.02 (0.82-1.26)	1.03 (0.81-1.28)	1.03 (0.81-1.30)
AST levels					
CKD incidence (KDIGO criteria)					
AST non-elevated	1181	1.00 (Reference)	1.00 (Reference)	1.00 (Reference)	1.00 (Reference)
AST elevated	341	**1.55 (1.12-2.13)**	**1.64 (1.17-2.26)**	**1.66 (1.18-2.31)**	**1.69 (1.19-2.37)**
Albuminuria					
AST non-elevated	1181	1.00 (Reference)	1.00 (Reference)	1.00 (Reference)	1.00 (Reference)
AST elevated	341	**1.59 (1.11-2.24)**	**1.78 (1.24-2.53)**	**1.83 (1.27-2.60)**	**1.80 (1.24-2.58)**
eGFR					
AST non-elevated	1181	1.00 (Reference)	1.00 (Reference)	1.00 (Reference)	1.00 (Reference)
AST elevated	341	1.01 (0.89-1.13)	1.01 (0.89-1.13)	1.01 (0.88-1.13)	1.01 (0.88-1.14)

Results based on robust Poisson regression. Predictor =GGT reference range or ALT or AST, Outcome=CKD or albuminuria or eGFR. IRR= incidence rate ratio with 95% confidence interval. CKD is defined based on the race-free CKD-EPI 2021 equation. Albuminuria categorized as ACR < 3 and ≥3. eGFR categorized as < 60 and ≥ 60. Categorizations: GGT normal range (less 55 males and 38 females), elevated GGT (greater 55 for males and 38 for females). ALT non elevated (<40), elevated (≥40). AST non elevated (<40), elevated (≥40). ***Model 1****= unadjusted.* ***Model 2*** *= adjusted for age and sex.* ***Model 3*** *= model 2 + education.* ***Model 4****= Model 3 + smoking, physical activity, alcohol consumption, follow-up time, geographical location, obesity, diabetes, change (Δ) in markers, uric acid, and hypertension*

Interaction of Liver biomarkers and Demographic Factors

Age, sex, education level, and geographical location were examined as potential modifiers of the relationship between liver biomarkers and CKD outcomes. GGT levels exhibited a significant interaction with age [p=0.049], sex [0.004] and geographical location. Participants from urban [p=0.023] and Amsterdam settings [p=0.046] had a higher CKD risk compared to their rural counterparts. The interaction between ALT or AST and demographic factors, however, was not significant.

Supplementary Table S3: Interaction between baseline liver biomarkers and demographic factors in the relationship with CKD

	GGT		ALT		AST	
**Liver biomarkers**	**IRR(95% CI)**	**p-values**	**IRR(95% CI)**	**p-values**	**IRR(95% CI)**	**p-values**
**Age**						
Biomarker * Age	**1.01 (1.01-1.01)**	**0.049**	0.99 (0.99-1.01)	0.875	0.99 (0.99-1.01)	0.682
Sex						
Biomarker * Males	1.00 (Reference)	Reference	1.00 (Reference)	Reference	1.00 (Reference)	Reference
Biomarker * Females	**1.01 (1.01-1.01)**	**0.004**	1.01 (0.98-1.01)	0.957	1.01 (0.99-1.01)	0.414
**Education**						
Biomarker * No education	1.00 (Reference)	Reference	1.00 (Reference)	Reference	1.00 (Reference)	Reference
Biomarker * Low education	1.01 (0.99-1.01)	0.129	1.01 (0.97-1.02)	0.659	0.99 (0.97-1.02)	0.842
Biomarker * Intermediate education	0.99 (0.98-1.01)	0.147	0.98 (0.93-1.01)	0.117	0.99 (0.96-1.01)	0.083
Biomarker * High education	1.02 (0.97-1.06)	0.186	1.01 (0.95-1.05)	0.268	1.01 (0.94-1.06)	0.392
**Geographical location**						
Biomarker * Rural Ghanaians	1.00 (Reference)	Reference	1.00 (Reference)	Reference	1.00 (Reference)	Reference
Biomarker * Urban Ghanaians	**1.01 (1.01-1.01)**	**0.023**	1.01 (0.98-1.03)	0.471	0.99 (0.97-1.01)	0.942
Biomarker * Amsterdam Ghanaians	**1.01 (1.01-1.01)**	**0.046**	1.01 (0.98-1.02)	0.781	0.99 (0.96-1.01)	0.761

*Results based on robust Poisson regression. Predictor = GGT or ALT or AST *Age or sex or education or location, Outcome=CKD. Male sex, no education and rural Ghanaians were reference groups. CKD is defined based on the race-free CKD-EPI 2021 equation. IRR= incidence rate ratio with 95% confidence interval.*

Baseline characteristics of elevated FLI vs non-elevated FLI.

Participants with elevated Fatty Liver Index (FLI) differed significantly in demographics, anthropometry, and clinical characteristics compared to those with non-elevated FLI. Participants with elevated FLI were more likely to reside in urban (49.0%) or Amsterdam (37.6%) areas compared to rural areas (13.4%) ($p < 0.001$). They were older (mean age 47 years), predominantly female (75.2%), and more likely to be unemployed or receiving social benefits (9.5%) compared to their non-elevated counterparts. Anthropometric measures such as BMI (31.8 vs. 24.2 kg/m²) and waist-hip ratio (0.95 vs. 0.89) were significantly higher in the elevated group ($p < 0.001$), indicating greater obesity and central adiposity. Elevated FLI participants also exhibited worse metabolic profiles, with higher triglycerides (1.22 vs. 0.84 mmol/L) and cholesterol (5.35 vs. 4.64 mmol/L) ($p < 0.001$ for both). They were also more likely to have hypertension (52.8% vs. 31.3%) and obesity (73.1% vs. 6.9%) ($p < 0.001$) (Appendix 4 Table).

Supplementary Table S4: Baseline characteristics of non-elevated vs elevated FLI participants

Variables	Overall N=1482	Non elevated FLI N= 1147	Elevated FLI N=335	p-values
Demographics				
Ghanaians in Rural, n (%)	538 (36.3)	493 (43.0)	45 (13.4)	<0.001
Ghanaians in Urban, n (%)	513 (34.6)	349 (30.4)	164 (49.0)	
Ghanaians in Amsterdam, n (%)	431 (29.1)	305 (26.6)	126 (37.6)	
**Age, mean (SD)**	46 (11)	45 (11)	47 (9)	0.002
**Sex, n (%)**				
Females	954 (64.4)	702 (61.2)	252 (75.2)	<0.001
Males	528 (35.6)	445 (38.8)	83 (24.8)	
**Education, n (%)**				
Lower vocational	534 (37.5)	415 (37.6)	119 (37.1)	0.7
Intermediate	196 (13.8)	157 (14.2)	39 (12.1)	
Higher vocational	68 (4.8)	50 (4.5)	18 (5.6)	
**Employment status, n (%)**				
Full time	427 (30.5)	345 (31.8)	82 (25.9)	<0.001
Part-time	719 (51.3)	585 (53.9)	134 (42.4)	
Social benefits	68 (4.9)	41 (3.6)	27 (8.5)	
Retired	15 (1.1)	10 (0.9)	5 (1.6)	
Unable to work	77 (5.5)	47 (4.3)	30 (9.5)	
Student	10 (0.7)	7 (0.6)	3 (0.9)	
Anthropometry information				
**BMI (kg/m2), median (IQR)**	25.7 (21.9-29.5)	24.2 (20.9-27.1)	31.8 (29.9-34.3)	<0.001
**Waist hip ratio, median (IQR)**	0.90 (0.86-0.94)	0.89 (0.85-0.93)	0.95 (0.91-0.98)	<0.001
Lifestyle information				
**Any alcohol consumption, n (%)**	554 (37.4)	438 (38.2)	116 (34.6)	0.2
**Smoking, n (%)**				
Present	33 (2.3)	30 (2.7)	3 (0.9)	0.081
Past	89 (6.3)	64 (5.8)	25 (7.9)	
**Physical activity, n (%)**				
Moderate	252 (19.6)	202 (20.3)	50 (17.3)	<0.001
High	739 (57.6)	592 (59.5)	147 (50.9)	
Laboratory information				
**Albuminuria, n (%)**	88 (6.0)	58 (5.1)	30 (9.0)	0.008
**Triglycerides (mmol/L), median (IQR)**	0.92 (0.69, 1.21)	0.84 (0.65, 1.10)	1.22 (0.93, 1.64)	<0.001
**Cholesterol(mmol/L), median (IQR)**	4.84 (4.07, 5.55)	4.65 (3.96, 5.41)	5.35 (4.63, 6.08)	<0.001
**Uric acid (µmol/L), median (IQR)**	292 (245, 351)	285 (237, 342)	320 (272, 379)	<0.001
**Urine albumin(mg/L), median (IQR)**	4.0 (4.0-4.0)	4.0 (4.0-4.0)	4.0 (4.0-10.9)	<0.001
**Urine creatinine(mmol/L), median (IQR)**	10 (6, 15)	10 (6, 14)	11 (7, 16)	0.001
**Albumin creatinine ratio(mg/mmol), median(IQR)**	0.53 (0.35, 0.88)	0.52 (0.35, 0.85)	0.62 (0.37, 0.99)	0.018

**eGFR, median (IQR)**	86 (75, 99)	86 (76, 100)	82 (73, 94)	<0.001
**FLI score, mean (SD)**	29 (12, 56)	24 (16)	77 (10)	<0.001
Underlying conditions				
**Hypertension, n (%)**	535 (36.1)	358 (31.2)	177 (52.8)	<0.001
**Diabetes, n (%)**	87 (5.9)	56 (4.9)	31 (9.3)	0.003
**Obesity, n (%)**	325 (21.9)	80 (7.0)	245 (73.1)	<0.001
Use of medication for the underlying health conditions				
**Hypertension medication, n (%)**	193 (13.0)	122 (10.6)	71 (21.2)	<0.001
**Diabetes medication, n (%)**	36 (2.4)	20 (1.7)	16 (4.8)	0.002

Data are presented as percentages, means (SDs), or median (interquartile range). Percentages are rounded to one decimal point and may not sum to 100%. % = Percentages. eGFR= Estimated glomerular filtration rate. BMI=body mass index. For p-values: Wilcoxon rank sum test; Pearson's Chi-squared test.

Supplementary Table S5: Exploratory association between FLI and FLI categorized with CKD outcomes six years later

**FLI score**	**Model 1 IRR(95% CI)**	**Model 2 IRR(95% CI)**	**Model 3 IRR(95% CI)**	**Model 4 IRR(95% CI)**
**FLI as a continuous predictor**				
FLI and CKD incidence (KDIGO criteria)	1.01 (0.99-1.01)	1.01 (0.99-1.01)	1.01 (0.99-1.01)	1.01 (0.99-1.01)
FLI and Albuminuria	**1.01 (1.01-1.01)**	**1.01 (1.01-1.01)**	1.01 (0.99-1.01)	**1.02 (1.01-1.03)**
FLI and eGFR	0.99 (0.99-1.01)	0.99 (0.99-1.01)	0.99 (0.99-1.01)	1.01 (0.99-1.01)
**FLI as a categorical predictor**				
CKD incidence (KDIGO criteria)				
Non elevated FLI	1.00 (Reference)	1.00 (Reference)	1.00 (Reference)	1.00 (Reference)
Elevated FLI	**1.63 (1.17-2.23)**	**1.52 (1.09-2.10)**	**1.55 (1.10-2.15)**	**1.92 (1.14-3.18)**
Albuminuria				
Non elevated FLI	1.00 (Reference)	1.00 (Reference)	1.00 (Reference)	1.00 (Reference)
Elevated FLI	**1.65 (1.16-2.33)**	**1.50 (1.05-2.12)**	**1.47 (1.02-2.08)**	**2.15 (1.24-3.68)**
eGFR				
Non elevated FLI	1.00 (Reference)	1.00 (Reference)	1.00 (Reference)	1.00 (Reference)
Elevated FLI	0.98 (0.87-1.11)	0.98 (0.87-1.11)	0.98 (0.86-1.11)	1.01 (0.82-1.21)

Results based on robust Poisson regression. Predictor =FLI, or FLI categorical, Outcome=CKD or Albuminuria or eGFR. CKD is defined based on the race-free CKD-EPI 2021 equation. Albuminuria categorized as ACR < 3 and ≥3. eGFR categorized as < 60 and ≥ 60. IRR= incidence rate ratio with 95% confidence interval. ***Model 1****= unadjusted.* ***Model 2*** *= adjusted for age and sex.* ***Model 3*** *= model 2 + education.* ***Model 4****= Model 3 + smoking, physical activity, alcohol consumption obesity, diabetes, uric acid, and hypertension.*

Interaction Between Baseline FLI and Demographic Factors

The interaction analysis showed no significant modifications of the relationship between FLI and CKD by age, sex, education, or geographical location. For age, the interaction term between FLI and age was not statistically significant (p = 0.467), indicating that the effect of FLI on CKD did not vary significantly between younger and older participants. Similarly, there was no significant interaction by sex (p = 0.224), suggesting the risk associated with elevated FLI was comparable between males and females. Educational attainment also did not modify the FLI-CKD association (p-values ranged from 0.268 to 0.731), nor did geographical location, with no differences observed between rural, urban, or Amsterdam-based participants (p = 0.316) (Appendix 4).

Supplementary Table S6: Interaction between baseline FLI and demographic factors in the relationship with CKD

**Markers**	**IRR(95% CI)**	**p-values**
Age		
FLI * Age	0.99 (0.99-1.01)	0.467
Sex		
FLI * Males	1.00 (Reference)	1.00 (Reference)
FLI * Females	0.99 (0.99-1.01)	0.224
Education		
FLI * No education	1.00 (Reference)	1.00 (Reference)
FLI * Low education	1.01 (0.99-1.01)	0.316
FLI * Intermediate education	0.99 (0.98-1.02)	0.731
FLI * High education	1.01 (0.97-1.05)	0.268
Geographical location		
FLI * Rural Ghanaians	1.00 (Reference)	1.00 (Reference)
FLI * Urban Ghanaians	1.01 (0.99-1.01)	0.332
FLI * Amsterdam Ghanaians	0.99 (0.97-1.01)	0.316

*Results based on robust Poisson regression. Predictor = FLI*Age or sex or education or location, Outcome=CKD. male sex, no education and rural Ghanaians were reference groups. CKD is defined based on the race-free CKD-EPI 2021 equation. IRR= incidence rate ratio with 95% confidence interval.*

Supplementary Table S7: Characteristics of all excluded participants

Variables	N or Mean or Median	% or SD or IQR
Baseline Characteristics		
Total numbers enrolled	346	
Ghanaians in rural areas, n (%)	14	4.0
Ghanaians in urban areas, n (%)	3	0.9
Ghanaians in Amsterdam The Netherlands, n (%)	329	95.1
Demographics		
**Age, mean (SD)**	46	12
**Sex, n (%)**		
Females	143	41.3
Males	203	58.7
**Education, n (%)**		
Lower vocational	121	38.8
Intermediate	81	26.0
Higher vocational	25	8.0
**Employment status, n (%)**		
Full time	132	42.9
Part-time	66	21.4
Social benefits	45	14.6
Retired	2	0.6
Unable to work	27	8.8
Student	10	3.2
Anthropometry information		
**BMI (kg/m2), median (IQR)**	27.5	24.3-30.3
**Waist hip ratio, median (IQR)**	0.90	0.84-0.95
Lifestyle information		
**Any alcohol consumption, n (%)**	20	76.9
**Smoking, n (%)**		
Present	14	4.5
Past	28	8.9
**Physical activity, n (%)**		
Moderate	6	24.0
High	15	60.0
Laboratory information		
**Triglycerides (mmol/L), median (IQR)**	0.62	0.47-0.89
**Cholesterol(mmol/L), median (IQR)**	4.85	4.20-5.57
**Uric acid (µmol/L), median (IQR)**	342	275-396
**Urine albumin(mg/L), median (IQR)**	4.0	4.0-5.0
**Urine creatinine(mmol/L), median (IQR)**	10	7-15
**Albumin creatinine ratio(mg/mmol), median (IQR)**	0.50	0.34-0.78
**eGFR, median (IQR)**	92	80-104
Underlying conditions		
**Hypertension, n (%)**	171	49.4
**Diabetes, n (%)**	30	8.7
**Obesity, n (%)**	98	28.4
Use of medication for the underlying health conditions		
**Hypertension medication, n (%)**	75	21.7
**Diabetes medication, n (%)**	12	3.5
Biomarkers of Liver cell damage		
**Gamma Glutamyl Transferase u/l, IQR, median (IQR)**		
Elevated	12	30.0
**Alanine Transaminase u/l, median (IQR)**		
Elevated	3	7.5
**Aspartate Transaminase u/l, median (IQR)**		
Elevated	10	25.0
Follow-up CKD outcomes		
**CKD incidence, n (%)**		
Yes	31	9.0
**Albuminuria, n (%)**		
Yes	19	5.7
**eGFR**		
Decreased (<60)	13	3.8

After removing duplicates, a total of 346 remained. Data are presented as percentages, means (SDs), or median (interquartile range). Percentages are rounded to one decimal point and may not sum to 100%. % = Percentages. eGFR= Estimated glomerular filtration rate. BMI=body mass index.

Supplementary Table S8: Baseline characteristics of included vs lost to follow-up

Variables	Overall N=4,222	Included N= 1,832	Lost to follow-up N=2,390	p-values
Demographics				
Ghanaians in Rural, n (%)	1,014 (24.0)	552 (30.1)	462 (19.3)	<0.001
Ghanaians in Urban, n (%)	1,356 (32.1)	516 (28.2)	840 (35.1)	
Ghanaians in Amsterdam, n (%)	1,852 (43.9)	764 (41.7)	1,088 (45.5)	
**Age, mean (SD)**	46 (12)	46 (11)	46 (13)	0.8
**Sex, n (%)**				
Females	2,684 (63.6)	1,157 (63.2)	1,527 (63.9)	0.6
Males	1,538 (36.4)	675 (36.8)	863 (36.1)	
**Education, n (%)**				
Lower vocational	1,425 (36.0)	655 (37.6)	770 (34.7)	0.2
Intermediate	639 (16.1)	281 (16.1)	358 (16.1)	
Higher vocational	212 (5.4)	93 (5.3)	119 (5.4)	
**Employment status, n (%)**				
Full time	1239 (30.2)	561 (32.7)	678 (28.3)	>0.9
Part-time	1710 (41.6)	785 (45.8)	925 (38.7)	
Social benefits	257 (6.26)	113 (6.6)	144 (6.02)	
Retired	65 (1.5)	17(0.9)	48 (2.0)	
Unable to work	289 (7.04)	104 (6.07)	185 (7.74)	
Student	60 (1.46)	20 (1.16)	40 (1.67)	
Anthropometry information				
**BMI (kg/m2), median (IQR)**	26.1 (22.5, 29.9)	26.0 (22.4, 29.7)	26.1 (22.5, 30.1)	0.2
**Waist hip ratio, median (IQR)**	0.90 (0.86, 0.95)	0.90 (0.85, 0.94)	0.90 (0.86, 0.95)	0.12
Lifestyle information				
**Any alcohol consumption, n (%)**	1,387 (36.2)	578 (38.2)	809 (34.9)	0.038
**Smoking, n (%)**				
Present	103 (2.6)	47 (2.7)	56 (2.6)	0.4
Past	290 (7.4)	117 (6.8)	173 (7.9)	
**Physical activity, n (%)**				
Moderate	612 (19.9)	258 (19.6)	354 (18.8)	<0.001
High	1738 (56.5)	754 (57.4)	984 (52.4)	
Laboratory information				
**Albuminuria, n (%)**	437 (10.5)	108 (5.9)	329 (14.2)	<0.001
**Triglycerides (mmol/L), median (IQR)**	0.86 (0.64, 1.20)	0.86 (0.64, 1.16)	0.86 (0.64, 1.22)	0.5
**Cholesterol(mmol/L), median (IQR)**	4.83 (4.07, 5.60)	4.84 (4.11, 5.55)	4.83 (4.03, 5.64)	>0.9
**Uric acid (µmol/L), median (IQR)**	295 (242, 357)	294 (245, 352)	296 (240, 361)	0.4
**Urine albumin(mg/L), median (IQR)**	4 (4, 9)	4 (4, 5)	4 (4, 11)	<0.001
**Urine creatinine(mmol/L), median (IQR)**	10 (6, 15)	10 (6, 15)	10 (6, 16)	0.2
**Albumin creatinine ratio(mg/mmol), median(IQR)**	0.54 (0.34, 0.93)	0.53 (0.35, 0.85)	0.55 (0.33, 1.01)	0.021
**eGFR, median (IQR)**	86 (75, 100)	86 (76, 100)	86 (74, 99)	0.002
Underlying conditions				
**Hypertension, n (%)**	1,782 (42.2)	710 (38.8)	1,072 (44.9)	<0.001
**Diabetes, n (%)**	349 (8.3)	117 (6.4)	232 (9.7)	<0.001
**Obesity, n (%)**	1,031 (24.5)	423 (23.1)	608 (25.5)	0.071
Use of medication for the underlying health conditions				
**Hypertension medication, n (%)**	1,211 (28.7)	272 (14.8)	939 (39.3)	<0.001
**Diabetes medication, n (%)**	152 (3.6)	48 (2.6)	104 (4.4)	0.003
**Biomarkers of liver cell damage**				
**GGT measurement U/L, median(IQR)**	30 (22, 42)	30 (22, 42)	30 (22, 42)	0.4
**ALAT measurement U/L, median(IQR)**	18 (14, 24)	19 (15, 25)	18 (14, 24)	0.026
**ASAT measurement U/L, median(IQR)**	31 (25, 38)	32 (26, 39)	30 (24, 38)	<0.001

Data are presented as percentages, means (SDs), or median (interquartile range). Percentages are rounded to one decimal point and may not sum to 100%. % = Percentages. eGFR= Estimated glomerular filtration rate. BMI=body mass index. For p-values: Wilcoxon rank sum test; Pearson's Chi-squared test.

Supplementary Table S9: Comparison of fully adjusted associations between baseline z-standardised liver biomarkers and CKD outcomes with and without inverse probability weighting (IPW).

Liver biomarkers	Model 4 (Primary) IRR (95% CI)	Model 4 + IPW IRR (95% CI)
**CKD incidence (KDIGO criteria)**		
GGT	**1.12 (1.01-1.23)**	**1.15 (1.03-1.26)**
ALT	**1.27 (1.04-1.50)**	**1.20 (1.00-1.39)**
AST	**1.20 (1.04-1.34)**	**1.17 (1.02-1.30)**
**Albuminuria**		
GGT	**1.27 (1.10-1.43)**	**1.22 (1.07-1.36)**
ALT	**1.26 (1.01-1.51)**	1.19 (0.97-1.40)
AST	**1.28 (1.08-1.49)**	**1.26 (1.06-1.45)**
**eGFR**		
GGT	0.99 (0.94-1.05)	1.00 (0.94-1.05)
ALT	0.99 (0.90-1.07)	0.99 (0.91-1.07)
AST	0.99 (0.92-1.06)	1.00 (0.93-1.06)

Results based on robust Poisson regression. Results are based on robust Poisson regression models. Liver biomarkers were z-standardised. CKD was defined according to KDIGO criteria using the race-free CKD-EPI 2021 equation. Albuminuria was categorized as ACR <3 vs ≥3 mg/mmol. Decreased eGFR was defined as <60 vs ≥60 mL/min/1.73m². Model 4 adjusted for age, sex, education, BMI, obesity, diabetes, hypertension, physical activity, smoking, alcohol consumption, and uric acid. IPW models (Model 4s) were additionally weighted using inverse probability weights derived from baseline predictors of follow-up status (age, sex, site, baseline eGFR, albuminuria, hypertension, and diabetes).

Supplementary Table S10: Association of liver- biomarkers composition with CKD incidence, albuminuria and decreased eGFR six years later.

**Liver-biomarkers composition vs CKD parameters**	**N**	**Model 1**<br><br>**IRR (95% CI)**	**Model 2**<br><br>**IRR (95% CI)**	**Model 3**<br><br>**IRR (95% CI)**
**CKD incidence and liver-biomarkers composition**				
No elevated	964	1.00 (Reference)	1.00 (Reference)	1.00 (Reference)
1 marker elevated	396	1.35 (0.95-1.90)	1.25 (0.88-1.77)	1.20 (0.82-1.73)
2 marker elevated	113	**2.46 (1.56-3.75)**	**2.64 (1.66-4.04)**	**2.41 (1.49-3.79)**
3 markers elevated	49	1.74 (0.78-3.37)	2.18 (0.96-4.27)	**2.47 (1.08-4.92)**
**Albuminuria and liver-biomarkers composition**				
No elevated	964	1.00 (Reference)	1.00 (Reference)	1.00 (Reference)
1 marker elevated	396	1.28 (0.87-1.85)	1.22 (0.83-1.78)	1.15 (0.76-1.71)
2 marker elevated	113	**2.33 (1.42-3.68)**	**2.63 (1.59-4.16)**	**2.25 (1.33-3.66)**
3 markers elevated	49	1.79 (0.75-3.60)	2.39 (0.99-4.89)	**2.73 (1.12-5.72)**
**eGFR and liver-biomarkers composition**				
No elevated	964	1.00 (Reference)	1.00 (Reference)	1.00 (Reference)
1 marker elevated	396	1.73 (0.74-3.87)	1.49 (0.64-3.34)	1.49 (0.64-3.34)
2 marker elevated	113	**3.64 (1.29-9.09)**	**3.09 (1.08-7.83)**	**3.09 (1.08-7.83)**
3 markers elevated	49	1.40 (0.07-6.97)	1.24 (0.06-1.11)	1.24 (0.06-1.11)

Results based on robust Poisson regression. Predictor =Liver-biomarkers composition. Outcome=CKD or albuminuria or eGFR. CKD is defined based on the race-free CKD-EPI 2021 equation. Albuminuria categorized as ACR < 3 and ≥3 mg/mmol. eGFR categorized as < 60 and ≥ 60. IRR= incidence rate ratio with 95% confidence interval. ***Model 1****= unadjusted.* ***Model 2*** *= adjusted for age or sex or both.* ***Model 3*** *= model 2 + smoking, physical activity, alcohol consumption, obesity, diabetes, uric acid, hypertension, and medication.*
